# Modifying the pre-pitch entry practices of professional soccer substitutes may contribute towards improved movement-related performance indicators on match-day: A case study

**DOI:** 10.1371/journal.pone.0232611

**Published:** 2020-05-05

**Authors:** Samuel P. Hills, Stephen Barrett, Matthew Hobbs, Martin J. Barwood, Jon N. Radcliffe, Carlton B. Cooke, Mark Russell

**Affiliations:** 1 School of Social and Health Sciences, Leeds Trinity University, Leeds, England, United Kingdom; 2 PlayerMaker, Hawley Wharf, London, England, United Kingdom; 3 GeoHealth Laboratory, Geospatial Research Institute, University of Canterbury, Christchurch, New Zealand; Nottingham Trent University, England, UNITED KINGDOM

## Abstract

Modifying a soccer substitute’s pre-pitch-entry activities may represent an opportunity to maximise physical performance and minimise injury-risk following match-introduction. Using a professional team that has previously participated in substitute profiling research, this follow-up case study investigated the effects of a modified match-day protocol that included substitutes; 1) performing a new pre-match warm-up alongside members of the starting team (as opposed to a separate substitute-only warm-up), 2) participating in a staff-led half-time rewarm-up (as opposed to player-led half-time activities), and 3) receiving ongoing education focusing on the efficacy of (re)warm-up activities. English Championship substitutes (n = 15) were monitored using Micro-electromechanical Systems during 13 matches incorporating the modified practices (35 observations). On an individual player basis, data were organised into bouts of warm-up activity (pre-pitch-entry) and five min epochs of match-play (post-pitch-entry). Linear mixed modelling assessed the influence of ‘bout’ and ‘epoch’, position, and scoreline. Substitutes performed 3±1 rewarm-up bouts∙player^-1^∙match^-1^ between kick-off and pitch-entry, which were shorter (-17.2 to -27.1 min) and elicited less distance (-696 to -1257 m) than the pre-match warm-up (p≤0.001). Compared with previous data, heightened absolute movement responses were observed during the pre-match and staff-led half-time (re)warm-ups, alongside greater relative distances covered during player-led activities performed between kick-off and pitch-entry. Whilst less distance (-10%) was covered during the second versus first five min period following match-introduction, values remained higher than previously reported. Between pitch-entry and the end of the match, the scoreline improved and worsened following 26% and 11% of substitutions, respectively; a favourable record compared with existing observations. Acknowledging the likely contribution from external factors, this case study reports heightened movement profiles and improved match scorelines when pre-pitch-entry practices were modified. Practitioners should note the potential influence of match-day activities on the physical responses of soccer substitutes and, if deemed necessary, consider adapting their pre-pitch-entry routines accordingly.

## Introduction

Although specific substitution regulations vary between competitions, soccer teams are permitted to replace a number of starting players during a match, on either a permanent or ‘rolling’ basis. For example, English Football League rules currently allow up to three substitutions to be made from a maximum of seven nominated players [1]. Aside from replacements enforced due to injury, coaches/managers typically introduce substitutes at half-time or during the second-half of match-play, often with the primary objectives of providing physical impetus and/or changing team tactics [2–5]. However, it is acknowledged that the use of substitutions may also reflect other motivations, such as decisions to replace players adjudged to be injured or underperforming, or a desire to allow playing time for youth players or those returning from injury [3, 5].

For outfield players who start the match on the pitch, progressive declines in indices of physical and technical performance are observed over the course of 90 min [6–8]. As the use of substitutions often represents a means by which coaches and managers seek to offset such negative responses via the introduction of ‘fresh legs’ [5], a substitute’s physical output may be an important indicator of match performance [2, 5]. Indeed, empirical evidence suggests that the execution of specific locomotor actions, such as the amount of high-speed running (HSR) performed by a player and/or team, represents a key performance indicator during professional soccer match-play [6, 9]. Notably, although players introduced as substitutes may typically exceed the relative total (TD) and/or HSR distances that they habitually adopt during the equivalent second-half period when the same individuals complete a whole match [2, 3, 6], they may be unable to surpass the movement responses that they would ordinarily produce during the first-half of matches in which they start [2, 3, 10]. Whilst team tactics, self-pacing strategies, and changes in other contextual factors (i.e., the match scoreline or the activities of other players) are likely to influence the running demands experienced during match-play [7, 11, 12], it is possible that these observations may partly reflect differences in the pre-pitch-entry preparations undertaken by substitutes compared with members of the starting eleven [3, 12]. In support, match-day strategies appear to vary markedly between teams and between individuals, with practitioners having noted the potential for sub-optimal pre-pitch-entry preparations to negatively influence team performance following a substitute’s introduction into a match [5].

Notwithstanding the benefits of an active pre-match warm-up to help starting players transition from a state of rest to a state of exercise [13–16], subsequent inactivity may induce physiological responses (e.g., decreases in body temperature) that could compromise muscular performance during high-intensity exercise performed thereafter, at least in thermoneutral environments [16, 17]. For this reason, half-time research has highlighted how extended periods of passive rest may not represent optimal preparation for the second-half of team sport match-play. Indeed, performing an active rewarm-up at half-time may help to attenuate body temperature declines, maintain physical performance, and potentially reduce the risk of injury when the second-half commences [15, 18–21]. As substitutes typically face lengthy delays (i.e., often ≥75–90 min) between the end of the pre-match warm-up and their introduction into a match [2, 3, 12], it is possible that the practices adopted during this time may have direct relevance to a player’s physical performance and/or injury-risk following pitch-entry.

Although substitutes awaiting introduction may perform short bouts of rewarm-up activity whilst the match is underway and potentially at half-time, much of the period between kick-off and pitch-entry is typically spent seated beside the pitch [12]. Although the efficacy of such practices for maximising match-performance and minimising injury-risk remains unclear, it has been suggested that the intensity of warm-up activity represents an important factor in determining the effectiveness of any preparatory strategy employed [13, 16, 22, 23]. For example, amongst team sport players, beneficial effects on repeated sprint ability have been observed following a warm-up incorporating actions conducted above, versus below, the anaerobic threshold [23]. Similarly, middle-distance runners produced improved 800 m running performance when prior warm-up exercise was modified from 300 m of striding to include an equidistant bout of combined striding and race-pace running [22]. In the only study to have profiled the pre-pitch-entry practices of soccer substitutes, players covered <2 m∙min^-1^ of HSR (defined as distance covered at a speed of ˃5.5 to ≤7 m∙s^-1^) during each bout of warm-up or rewarm-up activity performed, and recorded no sprinting (SPR; >7 m∙s^-1^) at any time prior to match-introduction [12]. Acknowledging that these reports were limited to players from a single club, and that other non-pitch-based activities (e.g., dynamic stretching or static cycling) may also have been performed, such observations highlight the need for further research in this area; a statement supported by applied practitioners [5]. Therefore, this follow-up case study aimed to profile the pre- and post-pitch-entry movement responses of professional soccer substitutes following modification of their pre-pitch-entry routine. Such information would add to the currently limited literature existing in relation to soccer substitutes and may aid practitioners seeking to improve the match-day preparations of partial-match soccer players.

## Methods

After receipt of ethical approval from the School of Social and Health Sciences Research Ethics Committee at Leeds Trinity University (SSHS-2019-003), fifteen outfield players (age: 26 ± 5 years, stature: 1.82 ± 0.05 m, body mass: 79.1 ± 5.8 kg) from a professional soccer club were monitored during 13 English Championship matches in which they entered the pitch as substitutes. From the sample consisting of eight midfielders (18 observations), three attackers (11 observations), and four defenders (six observations), a total of 35 individual player observations were yielded (2 ± 1 observations∙player^-1^, range: 1–6 observations∙player^-1^). Data from unused substitutes (i.e., members of the match-day squad who were not introduced onto the pitch during a match) were not included in the analyses [12]. All players were fully briefed about the risks and benefits of participation before providing written consent prior to data-collection, which took place during the 2018/2019 season.

Activity monitoring was conducted as per previous research [12], whereby substitutes’ movements were captured by 10 Hz Micro-electromechanical Systems (MEMS; S5, Optimeye, Catapult Innovations, Melbourne, Australia) worn between the scapulae and beneath the playing jersey in a vest designed to minimise movement artefacts. Notably, MEMS sampling at 10 Hz have demonstrated acceptable reliability (coefficient of variation; CV% = 2.0–5.3%) for measuring instantaneous velocity [24], whilst the specific models used have produced small-to-moderate typical errors of the estimate (1.87–1.95%) versus a radar gun when assessing sprinting speed [25]. The accelerometers within the devices have also demonstrated good intra (CV% = 0.9–1.1%) and inter-unit (CV% = 1.0–1.1) reliability in both laboratory and field test environments [26]. All players were familiar with this form of activity monitoring, with six of the included players having participated in substitute profiling research with same club previously [12]. Each individual wore the same MEMS unit in each match to avoid potential inter-unit variation.

The MEMS devices were activated according to the manufacturer’s guidelines ~30 min prior to the pre-match warm-up, and raw data files were exported post-match using proprietary software (Sprint 5.1.7, Catapult Innovations, Melbourne, Australia). The dependent variables of interest are outlined in Table 1, and were determined based upon speed, acceleration, and deceleration thresholds previously employed [12]. Individual data files were processed separately to allow organisation of pre-pitch-entry data into periods reflecting each bout of warm-up/rewarm-up activity performed, and the grouping of post-pitch-entry data into five min epochs from the moment a player entered the pitch [12]. For each substitution, contextual information relating to match scoreline, playing position, and the timing of a player’s introduction, was also recorded.

**Table 1:** Operational definition for Micro-electrical Mechanical Systems (MEMS)-derived outcome variables.

Measurement	Variable	Definition
**Distance covered**	TD (m)	Total amount of distance covered by any means
	Relative TD (m∙min^-1^)	Total amount of distance covered per minute
	LSR (m)	Distance covered at a speed of ≤4 m∙s^-1^
	Relative LSR (m∙min^-1^)	Distance covered per minute at a speed of ≤4 m∙s^-1^
	MSR (m)	Distance covered at a speed of ˃4 to ≤5.5 m∙s^-1^
	Relative MSR (m∙min^-1^)	Distance covered per minute at a speed of ˃4 to ≤5.5 m∙s^-1^
	HSR (m)	Distance covered at a speed of ˃5.5 to ≤7 m∙s^-1^
	Relative HSR (m∙min^-1^)	Distance covered per minute at a speed of ˃5.5 to ≤7 m∙s^-1^
	SPR (m)	Distance covered at a speed of ˃7 m∙s^-1^
	Relative SPR (m∙min^-1^)	Distance covered per minute at a speed ˃7 m∙s^-1^
**Running speed**	Peak velocity (m∙s^-1^)	Highest running speed attained
**PL**	PL (AU)	Quantification of external workload: Square root of the summed rates of change in instantaneous velocity in each of the three (forwards, sideways, upwards) vectors, divided by a scaling factor of 100
	Relative PL (AU∙min^-1^)	PL accumulated over X number of minutes, divided by X number of minutes
	PL·m^-1^ (AU∙m^-1^)	PL accumulated over X number of metres, divided by X number of metres
**Acceleration/deceleration distance**	High-speed acceleration (m)	Distance covered whilst accelerating at >3 m∙s^−2^
	High-speed deceleration (m)	Distance covered whilst decelerating at <−3 m∙s^−2^
	Moderate-speed acceleration (m)	Distance covered whilst accelerating at ˃2 to ≤3 m∙s^−2^
	Moderate-speed deceleration (m)	Distance covered whilst decelerating at <−2 to ≥−3 m∙s^−2^
**Time**	Duration (min)	Length of time for any given period

AU: Arbitrary units, HSR: High-speed running, LSR: Low-speed running, MEMS: Micro-electrical Mechanical Systems, MSR: Moderate-speed running, PL: PlayerLoad^™^, SPR: Sprinting, TD: Total distance.

This case study profiled the movement responses of soccer substitutes following modification of their match-day pre-pitch-entry routine compared with that reported previously [12]. Modification to substitutes’ match-day preparations reflected the combined effects of; undertaking an amended pre-match warm-up alongside members of the starting team (compared with the isolated low-intensity substitute-only warm-ups adopted previously [12]), and performing an extended (~13 min) staff-led group rewarm-up on the pitch at half-time (compared with ~6 min of individual player-led half-time practices [12]). Moreover, as part of an educational programme at the club, all players were briefed prior to, and regularly throughout, the season regarding the importance of warm-up and rewarm-up activities for enhancing physical performance and potentially reducing the risk of injury. Such information was delivered during a staff presentation prior to the season, repeated informally throughout the season, and consolidated via the display of posters at the team training facility.

The pre-match warm-up began with jogging and activation drills, before players performed free passing sequences with an emphasis on movement. Approximately 10 min of moderate-paced change of direction drills and dynamic stretching followed, before the warm-up concluded with high-tempo acceleration and deceleration activities, close-quarter possession games, and tactical set plays. The half-time rewarm-up was also directed by members of staff and lasted for the full duration of half-time (i.e., ~13 min; excluding brief transition periods immediately following the end of the first-half and prior to the second-half commencing). During this period, substitutes performed ~10 min of dynamic activities across the pitch, followed by free moving and passing sequences. Due to competition regulations preventing team officials from leaving their own technical area whilst a match is in play [27], the timing, content, and duration of any rewarm-up activities undertaken during the first- or second-halves were self-directed by the individual players without direct input from club staff. That said, all squad members had received educational sessions regarding the role of (re)warm-up activity in enhancing physical performance and potentially reducing the risk of injury.

### Statistical analyses

Due to the nested nature of data sampled via repeated observations of individuals across multiple matches (i.e., players nested within matches), linear mixed modelling was used to assess changes in outcome variables over time. In all models, ‘match’ and ‘player’ were entered as random effects to allow for natural variation between individual players and matches. Time (i.e., ‘epoch’ or ‘bout’) was modelled as a fixed effect, with the first time-period (i.e., ‘initial warm-up’ for pre-pitch-entry data, and ‘0–5 min’ for post-pitch-entry data) representing the reference category for comparison [12]. Match scoreline at the time of pitch-entry (i.e., ‘winning’, ‘losing’, ‘drawing’) was added to the pre- and post-pitch-entry models as a fixed effect, whilst playing position (i.e., ‘attackers’, ‘midfielders’, ‘defenders’) was also included as a fixed effect in the post-pitch-entry analyses. For the fixed effect of position, midfielders were used as the baseline comparator, and situations in which a player entered the pitch with the team being ahead in a match (i.e., ‘winning’) was specified as the reference category for the scoreline variable [12]. Where significant fixed effects were identified, pairwise comparisons were performed using least square means tests to compare outcomes at each level of the fixed effect. Analyses were conducted in R Studio statistical software version 3.6.1 (2019-07-05) using the lme4 package [28]. Data are presented as mean ± standard deviation (SD) unless otherwise indicated, whilst magnitude of change is demonstrated by effect estimates with associated 95% confidence intervals (CI). To allow comment on the potential influence of the modified versus existing practices, non-statistical comparisons were drawn with previously published data recorded from the same club (i.e., responses prior to modification [12]).

## Results

The maximum allocation of three substitutions was used in 10 out of the 13 matches observed. Two further matches involved the use of two substitutes and there was a single instance in which only one replacement was made. On average, the first, second, and third substitutions occurred after 68.69 ± 10.79, 78.25 ± 6.63, and 86.40 ± 4.05 min of match-play, respectively. The reference team won nine, drew three, and lost one of the 13 matches profiled, scoring 30 and conceding 13 goals in total. In 24 of the 35 substitutions observed, a player entered the pitch when their team was ahead in terms of match scoreline. There were four instances in which a substitute was introduced when the team was losing, and the remaining seven substitutions were made when the match scores were level. The mean scoreline at the time of pitch-entry was 2 ± 1 goals scored and 1 ± 1 goal conceded by the reference team. On nine occasions (26%), the team goal differential (i.e., goals scored minus goals conceded) improved during the time between a substitution being made and the end of the match. The goal differential became less favourable following four of the substitutions (11%) and had not changed by the end of the match (i.e., the same number of goals were scored by each team) in 22 instances (63%).

### Pre-pitch-entry responses

Substitutes performed an initial pre-match warm-up of ~30 min in duration, before completing 3 ± 1 bouts∙player∙match^-1^ of rewarm-up activity (range: 1–5 bouts∙player∙match^-1^) between kick-off and pitch-entry. The mean number of rewarm-ups performed independently during the first-half and second-half was 1 ± 1 bouts∙player∙match^-1^ (range: 0–2 bouts∙player∙match^-1^) and 1 ± 1 bouts∙player∙match^-1^ (range: 0–3 bouts∙player∙match^-1^), respectively. All participants in this case study performed a staff-led group rewarm-up of ~13 min in duration during half-time in each match.

Table 2 provides movement data relating to the pre-pitch-entry activities undertaken, whilst Table 3 indicates effect estimates and 95% CIs for rewarm-ups when compared with the initial warm-up. To allow broad comparison, previously published data from the same club is also presented in Table 2. Each rewarm-up was shorter, while eliciting less absolute TD and lower PlayerLoad^™^ (PL) values compared with the initial warm-up (all p ≤0.001). Less absolute low-speed running distance (LSR) was also observed during all rewarm-ups compared with the initial warm-up, alongside reductions in distance covered whilst accelerating or decelerating at moderate-speed, and distances covered whilst accelerating at high-speed (all p ≤0.05). Absolute moderate-speed running distance (MSR), and distance covered while decelerating at high-speed, were lower than the initial warm-up during all rewarm-ups except for the fourth rewarm-up performed independently (i.e., individual player-led) whilst a match was underway (all p ≤0.05). Expressed relative to bout duration (i.e., m∙min^-1^), and excluding the single instance of a fourth self-directed rewarm-up, substitutes covered more relative TD and LSR during all rewarm-ups (all p ≤0.001) compared with the initial warm-up. Higher relative PL values were observed during all rewarm-ups except for the fourth independent rewarm-up and the staff-led half-time rewarm-up (all p ≤0.05). With regards to HSR and SPR distances and compared with the initial warm-up, absolute values were higher during half-time (all p≤0.05). The initial warm-up also elicited less relative HSR compared with substitutes’ second player-led rewarm-up, and less relative SPR than the half-time rewarm-up (all p ≤0.05). Peak velocity remained below initial warm-up values during all rewarm-ups except for the fourth player-led rewarm-up (all p ≤0.001), whereas the peak velocity attained during the half-time rewarm-up was similar to the initial warm-up.

**Table 2:** Descriptive statistics for physical performance variables for substitutes prior to pitch-entry, including published data recorded from the same soccer club during the preceding season [12].

Variable		Initial warm-up		RWU1		Half-time rewarm-up		RWU2		RWU3		RWU4	
		(*n = 32*)	*Hills et al. [12], (n = 35)*	(*n = 25 first-half*, *n = 6 second-half*)	*Hills et al. [12], (n = 34 first-half, n = 1 second-half)*	(*n = 35*)	*Hills et al. [12], (n = 27)*	(*n = 15 first-half*, *n = 8 second-half*)	*Hills et al. [12], (n = 6 first-half, n = 22 second-half)*	(*n = 3 second-half*)	*Hills et al. [12], (n = 1 first-half, n = 7 second-half)*	(*n = 1 second-half*)	*Hills et al. [12], (n = 2 second-half)*
**Duration**	(min)	30.15 ± 4.40	*26*.*25 ± 2*.*43*	4.99 ± 1.87 ^b^	*6*.*51 ± 2*.*39* ^b^	12.99 ± 2.37 ^b^	*5*.*51 ± 2*.*31* ^b^	5.08 ± 4.07 ^b^	*5*.*96 ± 3*.*74* ^b^	2.94 ± 1.72 ^b^	*3*.*14 ± 1*.*68* ^b^	10.93 ± 0.00 ^b^	*3*.*23 ± 0*.*39* ^b^
**TD**	Absolute (m)	1498 ± 168	*992 ± 218*	337 ± 96 ^b^	*386 ± 143* ^b^	800 ± 183 ^b^	*423 ± 170* ^b^	454 ± 315 ^b^	*428 ± 286* ^b^	233 ± 136 ^b^	*229 ± 93* ^b^	495 ± 0 ^b^	*321 ± 44* ^b^
	Relative (m∙min^-1^)	50.3 ± 7.3	*37*.*9 ± 7*.*8*	71.8 ± 19.4 ^b^	*64*.*3 ± 23*.*5* ^b^	62.2 ± 11.6 ^a^	*83*.*0 ± 30*.*3* ^b^	107.5 ± 34.8 ^b^	*80*.*2 ± 28*.*9* ^b^	92.0 ± 41.8 ^b^	*89*.*3 ± 40*.*2* ^b^	45.3 ± 0	*99*.*5 ± 1*.*6* ^b^
**LSR**	Absolute (m)	1418 ± 158	*963 ± 210*	315 ± 98 ^b^	*369 ± 131* ^b^	748 ± 161 ^b^	*394 ± 159* ^b^	412 ± 307 ^b^	*378 ± 259* ^b^	216 ± 135 ^b^	*198 ± 100* ^b^	434 ± 0 ^b^	*280 ± 45* ^b^
	Relative (m∙min^-1^)	47.7 ± 6.9	*36*.*8 ± 7*.*5*	66.5 ± 16.9 ^b^	*61*.*1 ± 19*.*8* ^b^	58.1 ± 10.1^a^	*76*.*1 ± 22*.*9* ^b^	93.3 ± 24.9 ^b^	*70*.*7 ± 25*.*6* ^b^	81.7 ± 37.7 ^b^	*72*.*3 ± 28*.*1* ^b^	39.7 ± 0	*86*.*5 ± 3*.*4* ^b^
**MSR**	Absolute (m)	74 ± 31	*15 ± 31*	19 ± 25 ^b^	*15 ± 22*	37 ± 29 ^b^	*18 ± 28*	40 ± 32 ^b^	*42 ± 39* ^b^	17 ± 9 ^b^	*27 ± 26*	61 ± 0	*37 ± 6*
	Relative (m∙min^-1^)	2.5 ± 1.1	*0*.*6 ± 1*.*2*	4.8 ± 7.4	*2*.*9 ± 5*.*5*	2.9 ± 2.1	*4*.*5 ± 8*.*7* ^a^	12.6 ± 13.7 ^b^	*8*.*6 ± 9*.*3* ^b^	10.3 ± 12.1	*14*.*7 ± 18*.*0* ^b^	5.6 ± 0	*11*.*3 ± 0*.*6* ^b^
**HSR**	Absolute (m)	5 ± 8	*1 ± 4*	2 ± 6	*2 ± 6*	15 ± 20 ^b^	*3 ± 6*	2 ± 6	*6 ± 10* ^a^	0 ± 0	*3 ± 5*	0 ± 0	*5 ± 7*
	Relative (m∙min^-1^)	0.1 ± 0.2	*0*.*0 ± 0*.*1*	0.6 ± 1.4	*0*.*3 ± 1*.*0*	1.1 ± 1.4	*0*.*5 ± 1*.*3*	1.4 ± 1.4 ^a^	*0*.*8 ± 1*.*2* ^a^	0 ± 0	*1*.*9 ± 3*.*9* ^b^	0 ± 0	*1*.*7 ± 2*.*4* ^a^
**SPR**	Absolute (m)	0 ± 0	*0 ± 0*	0 ± 0	*0 ± 0*	1 ± 2 ^a^	*0 ± 0*	0 ± 0	*0 ± 0*	0 ± 0	*0 ± 0*	0 ± 0	*0 ± 0*
	Relative (m∙min^-1^)	0.0 ± 0.0	*0*.*0 ± 0*.*0*	0.0 ± 0.0	*0*.*0 ± 0*.*0*	0.1 ± 0.2 ^a^	*0*.*0 ± 0*.*0*	0.0 ± 0.0	*0*.*0 ± 0*.*0*	0.0 ± 0.0	*0*.*0 ± 0*.*0*	0.0 ± 0.0	*0*.*0 ± 0*.*0*
**Peak Velocity**	(m∙s^-1^)	5.4 ± 0.4	*n/a*	4.6 ± 1.0 ^b^	*n/a*	5.7 ± 0.9	*n/a*	4.9 ± 0.7 ^a^	*n/a*	4.5 ± 0.1 ^a^	*n/a*	4.8 ± 0	*n/a*
**PL**	Absolute (AU)	168.04 ± 20.91	*127*.*64 ± 24*.*10*	33.99 ± 9.37 ^b^	*38*.*54 ± 12*.*56* ^b^	84.27 ± 19.73 ^b^	*40*.*19 ± 19*.*29* ^b^	39.93 ± 22.72 ^b^	*42*.*50 ± 27*.*31* ^b^	20.02 ± 13.49 ^b^	*20*.*54 ± 9*.*26* ^b^	55.03 ± 0.00 ^b^	*30*.*27 ± 1*.*87* ^b^
	Relative (AU∙min^-1^)	5.64 ± 0.86	*4*.*88 ± 0*.*90*	7.27 ± 1.96 ^a^	*6*.*58 ± 2*.*79* ^b^	6.55 ± 1.34	*7*.*54 ± 2*.*05* ^b^	9.97 ± 3.58 ^b^	*7*.*90 ± 2*.*77* ^b^	9.01 ± 4.02 ^a^	*7*.*82 ± 3*.*72* ^b^	5.04 ± 0.00	*9*.*42 ± 0*.*55* ^b^
_ACC_dist	High (m)	11 ± 4	*2 ± 2*	1 ± 1 ^b^	*1 ± 1* ^*a*^	3 ± 3 ^b^	*2 ± 3*	2 ± 3 ^b^	*1 ± 4*	1 ± 2 ^b^	*1 ± 1*	2 ± 0 ^a^	*1 ± 0*
	Moderate (m)	23 ± 5	*11 ± 6*	4 ± 4 ^b^	*3 ± 3* ^b^	9 ± 5 ^b^	*5 ± 4*	6 ± 5 ^b^	*6 ± 7*	3 ± 2 ^b^	*2 ± 1*	9 ± 0 ^a^	*7 ± 1*
_DEC_dist	High (m)	3 ± 2	*0 ± 1*	0 ± 1 ^b^	*0 ± 1*	1 ± 1 ^b^	*0 ± 1*	1 ± 2 ^b^	*1 ± 1*^b^	0 ± 0 ^b^	*0 ± 0*	1 ± 0	*0 ± 0*
	Moderate (m)	10 ± 3	*3 ± 2*	2 ± 2 ^b^	*1 ± 2*	3 ± 3 ^b^	*2 ± 2*	3 ± 3 ^b^	*4 ± 4*^b^	0 ± 1 ^b^	*1 ± 1*	2 ± 0 ^a^	*2 ± 1*

_ACC_dist: Acceleration distance, AU: Arbitrary units, _DEC_dist: Deceleration distance, HSR: High-speed running, LSR: Low-speed running, MSR: Moderate-speed running, n/a: Not applicable, PL: PlayerLoad^™^, RWU: Self-directed rewarm-up, SPR: Sprinting, TD: Total distance

^a^ different from initial warm-up at p ≤0.05 level (within-study comparison)

^b^ different from initial warm-up at p ≤0.001 level (within-study comparison). Descriptive statistics from Hills et al. [12] are provided for illustrative purposes.

**Table 3:** Magnitude of change relative to initial warm-up values in physical performance variables for substitutes prior to pitch-entry.

Variable		Initial warm-up (*n = 32*)	RWU1 (*n = 25 first-half*, *n = 6 second-half*)	Half-time rewarm-up (*n = 35*)	RWU2 (*n = 15 first-half*, *n = 8 second-half*)	RWU3 (*n = 3 second-half*)	RWU4 (*n = 1 second-half*)	Scoreline effects
**Duration**	(min)	REF	-25.16 (-26.74 to -23.58) ^b^	-17.16 (-18.70 to 16.62) ^b^	-25.10 (-26.82 to -23.38) ^b^	-27.13 (-30.95 to -23.32) ^b^	-19.15 (-25.54 to -12.75) ^b^	None
**TD**	Absolute (m)	REF	-1160.90 (-1251.02 to -1070.74) ^b^	-695.89 (-783.34 to -608.66) ^b^	-1044.51 (-1143.02 to -945.83) ^b^	-1256.74 (-1479.38 to -1034.67) ^b^	-990.11 (-1365.96 to-615.07) ^b^	None
	Relative (m∙min^-1^)	REF	21.62 (12.06 to 31.12) ^b^	11.95 (2.68 to 21.16) ^a^	57.05 (46.66 to 67.42) ^b^	42.28 (19.01 to 65.49) ^b^	-2.51 (-42.38 to 37.01)	None
**LSR**	Absolute (m)	REF	-1102.74 (-1188.95 to -1016.53) ^b^	-669.50 (-753.31 to -586.05) ^b^	-1005.28 (-1099.29 to -910.97) ^b^	-1210.65 (-1423.33 to -998.99) ^b^	-990.01 (-1346.97 to -634.01) ^b^	None
	Relative (m∙min^-1^)	REF	18.95 (11.44 to 26.39) ^b^	10.65 (3.36 to 17.85) ^a^	45.38 (37.23 to 53.50) ^b^	34.43 (16.21 to 52.65) ^b^	-5.45 (-36.72 to 25.53)	None
**MSR**	Absolute (m)	REF	-55.19 (-68.17 to -42.21) ^b^	-38.04 (-50.60 to -25.45) ^b^	-33.44 (-47.65 to -19.25) ^b^	-59.70 (-91.81 to -27.68) ^b^	-16.26 (-70.27 to 37.69)	LO<WI*
	Relative (m∙min^-1^)	REF	2.30 (-1.94 to 5.79)	0.34 (-3.05 to 3.74)	10.45 (6.66 to 14.25) ^b^	7.55 (-0.92 to 16.03)	2.88 (-11.30 to 17.07)	None
**HSR**	Absolute (m)	REF	-2.35 (-7.64 to 2.97)	9.90 (4.78 to 15.04) ^b^	-2.48 (-8.29 to 3.38)	-3.59 (-16.96 to 9.66)	-2.77 (-25.30 to 19.61)	None
	Relative (m∙min^-1^)	REF	0.31 (-0.65 to 1.29)	0.82 (-0.11 to 1.77)	1.11 (0.04 to 2.19) ^a^	0.16 (-2.30 to 2.61)	0.31 (-3.85 to 4.45)	None
**SPR**	Absolute (m)	REF	<0.01 (-0.57 to 0.57)	0.65 (0.10 to 1.21) ^a^	0.01 (-0.61 to 0.63)	-0.05 (-1.45 to 1.33)	-0.05 (-2.38 to 2.27)	None
	Relative (m∙min^-1^)	REF	<0.01 (-0.04 to 0.04)	<0.01 (0.01 to 0.08) ^a^	<0.01 (-0.04 to 0.04)	<0.01 (-0.10 to 0.10)	<0.01 (-0.17 to 0.16)	None
**Peak Velocity**	(m∙s^-1^)	REF	-0.81 (-1.15 to -0.46) ^b^	0.26 (-0.07 to 0.60)	-0.43 (-0.81 to -0.05) ^a^	-0.89 (-1.77 to -0.02) ^a^	-0.54 (-2.02 to 0.92)	None
**PL**	Absolute (AU)	REF	-134.39 (-142.30 to -126.43) ^b^	-83.77 (-91.44 to -76.10) ^b^	-128.01 (-136.75 to -119.25) ^b^	-146.29 (-166.29 to -126.26) ^b^	-114.37 (-148.19 to -80.52) ^b^	None
	Relative (AU∙min^-1^)	REF	1.63 (0.65 to 2.61) ^a^	0.91 (-0.04 to 1.86)	4.34 (3.26 to 5.40) ^b^	3.40 (1.00 to 5.81) ^a^	-0.42 (-4.46 to 3.62)	None
_ACC_dist	High (m)	REF	-10.57 (-11.89 to -9.25) ^b^	-7.69 (-8.97 to -6.41) ^b^	-9.30 (-10.75 to -7.87) ^b^	-10.65 (-13.87 to -7.44) ^b^	-9.06 (-14.54 to -3.61) ^a^	None
	Moderate (m)	REF	-19.78 (-21.95 to -17.61) ^b^	-14.02 (-16.12 to -11.91) ^b^	-17.37 (-19.72 to -15.01) ^b^	-21.17 (-26.38 to -15.95) ^b^	-14.82 (-23.58 to -5.98) ^a^	LO<WI* LO<DR^†^
_DEC_dist	High (m)	REF	-2.76 (-3.42 to -2.11) ^b^	-2.61 (-3.25 to -1.98) ^b^	-1.91 (-2.62 to -1.21) ^b^	-3.28 (-4.87 to -1.68) ^b^	-2.28 (-4.93 to 0.38)	LO<WI*
	Moderate (m)	REF	-7.64 (-8.92 to -6.35) ^b^	-6.97 (-8.21 to -5.72) ^b^	-6.17 (-7.59 to -4.78) ^b^	-9.79 (-12.95 to -6.66) ^b^	-8.10 (-13.37 to -2.84) ^a^	LO<WI*

_ACC_dist: Acceleration distance, AU: Arbitrary units, DR: Scores level at the time of pitch-entry, _DEC_dist: Deceleration distance, HSR: High-speed running, LO: Team losing at the time of pitch-entry, LSR: Low-speed running, MSR: Moderate-speed running, PL: PlayerLoad^™^, REF: Reference category, RWU: Self-directed rewarm-up, SPR: Sprinting, TD: Total distance, WI: Team winning at the time of pitch-entry, #_ACC_: Number of accelerations, #_DEC_: Number of decelerations

^a^ different from initial warm-up at p ≤0.05 level

^b^ different from initial warm-up at p ≤0.001 level.

*: Significant effect at p ≤0.05 level

†: Significant effect at the p ≤0.016 level. Data are reported as effect estimates (95% CI).

Pairwise contrasts revealed that the staff-led half-time group rewarm-up was longer in duration (1.98 to 7.94 min) and elicited greater absolute TD (313.79 to 578.42 m), LSR (320.51 to 541.14 m), and PL (30.61 to 62.53 AU) values compared with all player-led rewarm-ups, except for the fourth (all p ≤0.003). In addition, substitutes during the half-time rewarm-up performed more absolute HSR (12.24 to 12.37 m) and attained a higher peak velocity (0.69 to 1.07 m·s^-1^) compared with the first and second independent rewarm-ups, whilst also covering greater high- (2.89, CI: 0.92 to 4.84 m) and moderate-speed (5.76, CI: 2.53 to 8.99 m) acceleration distances than during the first player-led rewarm-up only (all p ≤0.003). Relative values for TD (45.10, CI: 29.63 to 60.59 m·min^-1^), LSR (34.74, CI: 22.61 to 46.86 m·min^-1^), MSR (10.11, CI: 4.45 to 15.78 m·min^-1^), and PL (3.42, CI: 1.83 to 5.02 AU·min^-1^) were higher for players’ second unaccompanied rewarm-up compared with the half-time rewarm-up (all p ≤0.003). With regards to comparisons between player-led rewarm-ups performed whilst a match was underway (i.e., not including staff-led activities performed prior to kick-off or at half-time), substitutes covered less absolute MSR (-21.76, CI: -43.40 to -0.14 m·min^-1^), and recorded lower values for relative TD (-35.43, CI: -51.28 to -19.59 m·min^-1^), LSR (-26.43, CI: -38.83 to -14.02 m·min^-1^), MSR (-8.16, CI: -13.96 to -2.35 m·min^-1^), and PL (-2.71, CI: -4.34 to -1.08 AU·min^-1^) during the first independent rewarm-up compared with the second (all p ≤0.05).

Match scoreline at the time of pitch-entry influenced some, but not all, of the outcome variables profiled (Table 3). When the reference team was losing at the moment of a player’s introduction, substitutes performed less absolute MSR (-21.78, CI: -38.78 to -4.59 m), covered smaller distances whilst decelerating at high- (-0.85, CI: -1.58 to -0.11 m) and moderate-speed (-2.56, CI: -4.07 to -1.07 m), and accumulated less distance whilst accelerating at moderate-speed (-4.23, CI: -6.63 to -1.80 m) per rewarm-up, compared with when the team was winning (all p ≤0.05). On a per rewarm-up basis, substitutes also covered less moderate-speed acceleration distance (-4.28, CI: -7.85 to -0.70 m) when their team was losing at the time of pitch-entry, compared with when the match scores were level (p = 0.013).

### Post-pitch-entry responses

Once introduced onto the pitch, substitutes played an average of 17.90 ± 10.71 min and covered 2081 ± 1111 m·match^-1^ (Table 4). Tables 4 and 5 demonstrate that compared with the initial five min following a player’s introduction, TD and PL were lower for all subsequent match epochs (all p ≤0.05). In addition, MSR was less than 0–5 min values during all epochs except for those reflecting 25–30 min and 35–40 min post-pitch-entry, whereas LSR was lower during all, except the 30–35 min, epochs (all p ≤0.05). High-speed acceleration distance was less for 35–40 min compared with 0–5 min post-pitch-entry and moderate-speed acceleration distance was lower than 0–5 min between 10–15 min and 15–20 min (all p ≤0.05). Moderate-speed deceleration distance was less than 0–5 min values during the 10–15 min post-pitch-entry epoch only (p ≤0.05). Variables relating to the amount of HSR or SPR performed, peak velocity achieved, PL·m^-1^, and high-speed deceleration distance were maintained relative to the initial five min following pitch-entry. Moreover, no differences were observed between any other match epochs with respect any outcome measure profiled.

**Table 4:** Descriptive statistics for physical performance variables for substitutes from timing of pitch-entry to the end of match-play, including published data recorded from the same soccer club during the preceding season [12].

Variable		Whole bout	0–5 min		5–10 min		10–15 min		15–20 min		20–25 min		25–30 min		30–35 min		35–40 min	
			(*n = 33*)	*Hills et al. [12], (n = 33)*	(*n = 28*)	*Hills et al. [12], (n = 32)*	(*n = 18*)	*Hills et al. [12], (n = 30)*	(*n = 11*)	*Hills et al. [12], (n = 26)*	(*n = 9*)	*Hills et al. [12], (n = 19)*	(*n = 4)*	*Hills et al. [12], (n = 11)*	(*n = 3*)	*Hills et al. [12], (n = 7)*	(*n = 2*)	*Hills et al. [12], (n = 4)*
**Duration**	(min)	17.90 ± 10.71	5.00	*5*.*00*	5.00	*5*.*00*	5.00	*5*.*00*	5.00	*5*.*00*	5.00	*5*.*00*	5.00	*5*.*00*	5.00	*5*.*00*	5.00	*5*.*00*
**TD**	Absolute (m)	2081 ± 1111	646 ± 100	*599 ± 75*	579 ± 92 ^b^	*527 ± 66* ^b^	584 ± 80 ^a^	*527 ± 81* ^b^	546 ± 63 ^b^	*531 ± 59* ^b^	535 ± 97 ^b^	*527 ± 60* ^b^	488 ± 106 ^a^	*508 ± 72* ^b^	511 ± 32 ^a^	*507 ± 110* ^b^	462 ± 25 ^a^	*521 ± 56* ^a^
	Relative (m∙min^-1^)	121.8 ± 18.2	129.3 ± 20.0	*120*.*0 ± 14*.*8*	115.8 ± 18.5 ^b^	*105*.*3 ± 13*.*3* ^b^	116.7 ± 16.0 ^a^	*105*.*6 ± 16*.*5* ^b^	109.2 ± 12.6 ^b^	*106*.*0 ± 11*.*5* ^b^	107.0 ± 19.3 ^b^	*105*.*2 ± 11*.*8* ^b^	97.5 ± 21.1 ^a^	*101*.*7 ± 14*.*5* ^b^	102.1 ± 6.2 ^a^	*101*.*4 ± 22*.*1* ^b^	92.3 ± 4.9 ^a^	*104*.*2 ± 11*.*2* ^a^
**LSR**	Absolute (m)	1581 ± 897	474 ± 69	*438 ± 55*	431 ± 44 ^b^	*414 ± 49*	442 ± 64 ^a^	*414 ± 66*	422 ± 46 ^a^	*413 ± 7*	418 ± 48 ^a^	*402 ± 48* ^a^	387 ± 97 ^a^	*405 ± 51*	415 ± 59	*425 ± 80*	380 ± 71 ^a^	*431 ± 58*
	Relative (m∙min^-1^)	90.4 ± 9.5	94.9 ± 11.7	*87*.*6 ± 11*.*0*	86.2 ± 8.9 ^b^	*82*.*9 ± 9*.*8*	88.4 ± 12.8 ^a^	*82*.*8 ± 13*.*2*	84.3 ± 9.1 ^a^	*82*.*5 ± 9*.*3*	83.6 ± 9.6 ^a^	*80*.*5 ± 9*.*5* ^a^	77.4 ± 19.5 ^a^	*81*.*1 ± 10*.*3*	83.0 ± 11.8	*84*.*9 ± 16*.*0*	76.0 ± 14.1 ^a^	*86*.*1 ± 11*.*5*
**MSR**	Absolute (m)	336 ± 182	116 ± 43	*105 ± 34*	96 ± 44 ^a^	*72 ± 27* ^b^	96 ± 43 ^a^	*78 ± 38* ^b^	86 ± 50 ^a^	*78 ± 29* ^b^	78 ± 42 ^a^	*84 ± 36* ^a^	67 ± 30	*68 ± 33* ^b^	51 ± 32 ^a^	*58 ± 28* ^b^	59 ± 17	*72 ± 29* ^a^
	Relative (m∙min^-1^)	21.0 ± 8.7	23.3 ± 8.5	*20*.*9 ± 6*.*8*	19.2 ± 8.8 ^a^	*14*.*3 ± 5*.*4* ^b^	19.1 ± 8.5 ^a^	*15*.*5 ± 7*.*5* ^b^	17.2 ± 10.0 ^a^	*15*.*7 ± 5*.*8* ^b^	15.6 ± 8.4 ^a^	*16*.*8 ± 7*.*1* ^a^	13.3 ± 6.0	*13*.*6 ± 6*.*6* ^b^	10.3 ± 6.4 ^a^	*11*.*5 ± 5*.*5* ^b^	11.8 ± 3.4	*14*.*3 ± 5*.*8* ^a^
**HSR**	Absolute (m)	133 ± 76	48 ± 36	*51 ± 29*	40 ± 23	*31 ± 22* ^b^	37 ± 21	*28 ± 19* ^b^	31 ± 19	*30 ± 20* ^b^	31 ± 18	*36 ± 22* ^a^	28 ± 13	*24 ± 18* ^b^	27 ± 24	*20 ± 19* ^b^	9 ± 9	*18 ± 14* ^b^
	Relative (m∙min^-1^)	8.4 ± 4.8	9.5 ± 7.1	*10*.*1 ± 5*.*9*	8.1 ± 4.6	*6*.*2 ± 4*.*5* ^b^	7.4 ± 4.1	*5*.*7 ± 3*.*9* ^b^	6.2 ± 3.7	*6*.*1 ± 4*.*0* ^b^	6.2 ± 3.7	*7*.*1 ± 4*.*4* ^a^	5.7 ± 2.7	*4*.*9 ± 3*.*6* ^b^	5.5 ± 4.8	*3*.*9 ± 3*.*7* ^b^	1.8 ± 2.0	*3*.*7 ± 2*.*8* ^b^
**SPR**	Absolute (m)	32 ± 41	8 ± 13	*6 ± 10*	12 ± 25	*10 ± 15*	9 ± 16	*7 ± 11*	7 ± 20	*10 ± 12*	9 ± 18	*5 ± 10*	6 ± 5	*11 ± 14*	17 ± 29	*5 ± 9*	14 ± 19	*1 ± 2*
	Relative (m∙min^-1^)	2.0 ± 3.2	1.6 ± 2.6	*1*.*3 ± 1*.*9*	2.4 ± 4.9	*2*.*1 ± 3*.*1*	1.8 ± 3.1	*1*.*4 ± 2*.*1*	1.4 ± 4.0	*2*.*0 ± 2*.*5*	1.7 ± 3.6	*1*.*1 ± 2*.*0*	1.2 ± 1.1	*2*.*2 ± 2*.*8*	3.4 ± 5.9	*1*.*1 ± 1*.*8*	2.7 ± 3.8	*0*.*2 ± 0*.*3*
**Peak Velocity**	(m∙s^-1^)	7.3 ± 0.8	6.8 ± 0.8	*n/a*	6.7 ± 0.8	*n/a*	6.9 ± 0.7	*n/a*	6.6 ± 0.8	*n/a*	6.6 ± 1.0	*n/a*	7.1 ± 0.6	*n/a*	7.0 ± 1.6	*n/a*	7.1 ± 2.2	*n/a*
**PL**	Absolute (AU)	206.28 ± 108.74	65.92 ± 11.07	*61*.*21 ± 8*.*43*	59.07 ± 12.97 ^b^	*53*.*94 ± 6*.*80* ^b^	56.37 ± 10.85 ^b^	*52*.*78 ± 9*.*65* ^b^	54.49 ± 8.05 ^b^	*53*.*04 ± 8*.*17* ^b^	51.15 ± 12.61 ^b^	*52*.*90 ± 7*.*07* ^b^	49.06 ± 9.74 ^a^	*49*.*67 ± 6*.*28* ^b^	48.97 ± 7.58 ^a^	*46*.*31 ± 10*.*35* ^b^	43.79 ± 2.47 ^a^	*45*.*76 ± 10*.*38* ^b^
	Relative (AU∙min^-1^)	12.31 ± 2.57	13.18 ± 2.21	*12*.*25 ± 1*.*67*	11.61 ± 2.59 ^b^	*10*.*77 ± 1*.*39* ^b^	11.27 ± 2.17 ^b^	*10*.*59 ± 1*.*94* ^b^	10.90 ± 1.61 ^b^	*10*.*59 ± 1*.*61* ^b^	10.23 ± 2.52 ^b^	*10*.*55 ± 1*.*41* ^b^	9.81 ± 1.95 ^a^	*9*.*93 ± 1*.*25* ^b^	9.79 ± 1.52 ^a^	*9*.*26 ± 2*.*07* ^b^	8.76 ± 0.49 ^a^	*9*.*15 ± 2*.*08* ^b^
	Per metre (AU∙m^-1^)	0.10 ± 0.01	0.10 ± 0.01	*n/a*	0.10 ± 0.01	*n/a*	0.10 ± 0.01	*n/a*	0.10 ± 0.01	*n/a*	0.10 ± 0.01	*n/a*	0.10 ± 0.01	*n/a*	0.10 ± 0.01	*n/a*	0.10 ± 0.00	*n/a*
**_ACC_dist**	High (m)	19 ± 13	6 ± 3	*8 ± 3*	5 ± 3	*6 ± 3* ^a^	4 ± 2	*5 ± 3* ^b^	4 ± 4	*5 ± 3* ^b^	4 ± 3	*7 ± 4*	5 ± 5	*6 ± 2*	5 ± 7	*3 ± 3* ^b^	1 ± 1 ^a^	*6 ± 4*
	Moderate (m)	50 ± 28	16 ± 4	*16 ± 5*	14 ± 4	*13 ± 5* ^b^	13 ± 4 ^a^	*13 ± 4* ^b^	12 ± 5 ^a^	*13 ± 4* ^b^	13 ± 4	*14 ± 5* ^a^	13 ± 1	*13 ± 4* ^a^	11 ± 3	*10 ± 2* ^b^	10 ± 11	*11 ± 2* ^a^
**_DEC_dist**	High (m)	18 ± 12	5 ± 3	*5 ± 2*	5 ± 2	*4 ± 3* ^a^	4 ± 3	*3 ± 1* ^a^	4 ± 3	*4 ± 3*	4 ± 3	*5 ± 3*	2 ± 1	*4 ± 1* ^a^	4 ± 7	*3 ± 2* ^a^	5 ± 6	*3 ± 3* ^a^
	Moderate (m)	32 ± 17	10 ± 3	*10 ± 4*	9 ± 3	*8 ± 5*	8 ± 3 ^a^	*8 ± 3* ^a^	8 ± 5	*8 ± 3*	8 ± 6	*8 ± 4*	7 ± 2	*8 ± 3*	7 ± 2	*7 ± 4*	5 ± 5	*7 ± 4*

_ACC_dist: Acceleration distance, AU: Arbitrary units, _DEC_dist: Deceleration distance, HSR: High-speed running, LSR: Low-speed running, MSR: Moderate-speed running, n/a: Not applicable, PL: PlayerLoad^™^, SPR: Sprinting, TD: Total distance

^a^ different from 0–5 min at p ≤0.05 level (within-study comparison)

^b^ different from 0–5 min at p ≤0.001 level (within-study comparison). Descriptive statistics from Hills et al. [12] are provided for illustrative purposes.

**Table 5:** Magnitude of change from 0–5 min values in physical performance variables for substitutes from timing of pitch-entry to the end of match-play.

Variable		0–5 min (*n = 33*)	5–10 min (*n = 28*)	10–15 min (*n = 18*)	15–20 min (*n = 11*)	20–25 min (*n = 9*)	25–30 min (*n = 4*)	30–35 min (*n = 3*)	35–40 min (*n = 2*)	Position effects	Scoreline effects
**TD**	Absolute (m)	REF	-70.02 (-105.59 to -34.62) ^b^	-65.75 (-107.18 to -24.53) ^a^	-95.14 (-145.06 to -44.98) ^b^	-98.04 (-152.72 to -43.58) ^b^	-119.97 (-196.38 to -44.05) ^a^	-92.44 (-180.19 to -5.52) ^a^	-110.39 (-216.27 to -5.68) ^a^	ATT<MID** DEF<MID*	None
	Relative (m∙min^-1^)	REF	-14.01 (-21.12 to -6.93) ^b^	-13.15 (-21.44 to -4.91) ^a^	-19.03 (-29.01 to -9.00) ^b^	-19.61 (-30.54 to -8.72) ^b^	-23.97 (-39.24 to -8.79) ^a^	-18.49 (-36.03 to -1.11) ^a^	-22.08 (-43.25 to -1.14) ^a^	ATT<MID** DEF<MID*	None
**LSR**	Absolute (m)	REF	-45.24 (-69.02 to -21.62) ^b^	-37.80 (-65.38 to -10.11) ^a^	-53.06 (-86.51 to -19.56) ^a^	-55.65 (-92.21 to -19.20) ^a^	-74.30 (-125.27 to -23.41) ^a^	-54.89 (-113.11 to 3.46)	-70.40 (-140.82 to -0.33) ^a^	ATT<MID*	None
	Relative (m∙min^-1^)	REF	-9.05 (-13.80 to -4.32) ^b^	-7.56 (-13.08 to -2.02) ^a^	-10.61 (-17.30 to -3.91) ^a^	-11.13 (-18.44 to -3.84) ^a^	-14.86 (-25.05 to -4.68) ^a^	-10.97 (-22.62 to 0.69)	-14.07 (-28.16 to -0.06) ^a^	ATT<MID*	None
**MSR**	Absolute (m)	REF	-20.75 (-36.95 to -4.67) ^a^	-20.60 (-39.60 to -1.80) ^a^	-26.84 (-49.67 to -4.03) ^a^	-32.00 (-56.90 to -7.14) ^a^	-32.79 (-67.58 to 1.91)	-42.02 (-82.03 to -2.38) ^a^	-19.68 (-67.58 to 28.05)	ATT<MID** DEF<MID*	None
	Relative (m∙min^-1^)	REF	-4.15 (-7.39 to -0.93) ^a^	-4.12 (-7.92 to -0.36) ^a^	-5.37 (-9.93 to -0.80) ^a^	-6.40 (-11.38 to -1.43) ^a^	-6.56 (-13.52 to 0.38)	-8.40 (-16.41 to -0.48) ^a^	-3.93 (-13.54 to 5.61)	ATT<MID** DEF<MID*	None
**HSR**	Absolute (m)	REF	-7.17 (-18.67 to 4.38)	-8.62 (-22.17 to 4.72)	-15.78 (-32.97 to 0.64)	-13.36 (-31.05 to 4.41)	12.77 (-37.55 to 11.89)	-7.80 (-36.32 to 20.40)	27.54 (-61.68 to 6.53)	None	DR<WI*
	Relative (m∙min^-1^)	REF	-1.43 (-3.73 to 0.88)	-1.73 (-4.43 to 0.94)	-3.16 (-6.39 to 0.12)	-2.67 (-6.21 to 0.89)	-2.55 (-7.51 to 2.78)	-1.56 (-7.26 to 4.08)	-5.51 (-12.34 to 1.31)	None	DR<WI*
**SPR**	Absolute (m)	REF	2.71 (-4.86 to 10.52)	1.56 (-7.36 to 10.51)	0.26 (-10.55 to 11.12)	3.47 (-8.33 to 15.24)	1.19 (-15.37 to 17.61)	14.17 (-4.71 to 32.89)	9.02 (-13.60 to 31.58)	None	None
	Relative (m∙min^-1^)	REF	0.54 (-0.97 to 2.10)	0.31 (-1.47 to 2.10)	0.05 (-2.11 to 2.22)	0.69 (-1.67 to 3.04)	0.23 (-3.07 to 3.52)	2.83 (-0.94 to 6.58)	1.80 (-2.72 to 6.32)	None	None
**Peak Velocity**	(m∙s^-1^)	REF	-0.07 (-0.46 to 0.34)	0.13(-0.32 to 0.59)	-0.19 (-0.73 to 0.38)	-0.16 (-0.76 to 0.45)	0.31 (-0.56 to 1.11)	0.38 (-0.62 to 1.30)	0.30 (-0.86 to 1.46)	None	None
**PL**	Absolute (AU)	REF	-7.23 (-11.21 to -3.12) ^b^	-8.69 (-13.38 to -4.08) ^b^	-10.47 (-16.10 to -4.87) ^b^	-12.31 (-18.45 to -6.23) ^b^	-13.91 (-22.47 to –5.40) ^a^	-12.71 (-22.47 to -3.03) ^a^	13.61 (-25.34 to -1.98 ^a^	None	None
	Relative (AU∙min^-1^)	REF	-1.44 (-2.24 to -0.66) ^b^	-1.74 (-2.68 to -0.82) ^b^	-2.09 (-3.22 to -0.97) ^b^	-2.46 (-3.69 to -1.25) ^b^	-2.78 (-4.49 to -1.08) ^a^	-2.54 (-4.49 to -0.61) ^a^	-2.72 (-5.07 to -0.40) ^a^	None	None
	Per metre (AU∙m^-1^)	REF	<0.01	<0.01	<0.01	<0.01	<0.01	<0.01	<0.01	n/a	n/a
_ACC_dist	High (m)	REF	-0.24 (-1.66 to 1.18)	-1.36 (-3.00 to 0.28)	-0.86 (-2.83 to 1.12)	-1.31 (-3.46 to 0.85)	-0.95 (-3.94 to 2.04)	-0.01 (-3.44 to 3.44)	-4.62 (-8.80 to -0.46) ^a^	n/a	n/a
	Moderate (m)	REF	-2.09 (-4.10 to 0.08)	-3.49 (-5.82 to -1.18) ^a^	-3.35 (-6.17 to -0.58) ^a^	-2.60 (-5.70 to 0.41)	-2.69 (-6.92 to 1.54)	-3.82 (-8.79 to 0.96)	-4.15 (-10.07 to 1.69)	n/a	n/a
_DEC_dist	High (m)	REF	0.34 (-0.91 to 1.59)	-0.75 (-2.20 to 0.70)	-1.24 (-3.00 to 0.56)	-0.92 (-2.83 to 1.04)	-2.48 (-5.15 to 0.22)	0.21 (-2.84 to 2.28)	0.31 (-3.36 to 4.04)	n/a	n/a
	Moderate (m)	REF	-0.85 (-2.40 to 0.71)	-2.04 (-3.85 to -0.25) ^a^	-1.48 (-3.64 to 0.71)	-0.85 (-3.22 to 1.54)	-1.57 (-4.84 to 1.70)	-1.35 (-5.17 to 2.42)	-1.07 (-5.66 to 3.48)	DEF<MID*	LO<WI* DR<WI*

_ACC_dist: Acceleration distance, ATT: Attackers, AU: Arbitrary units, DR: Scores level at the time of pitch-entry, _DEC_dist: Deceleration distance, DEF: Defenders, HSR: High-speed running, LO: Team losing at the time of pitch-entry, LSR: Low-speed running, MID: Midfielders, MSR: Moderate-speed running, PL: PlayerLoad^™^, REF: Reference category, SPR: Sprinting, TD: Total distance, WI: Team winning at the time of pitch-entry

^a^ different from 0–5 min at p ≤0.05 level

^b^ different from 0–5 min at p ≤0.001 level

*: Significant effect at p ≤0.05 level

**: Significant effect at p ≤0.001 level. Data are reported as effect estimates (95% CI).

On a per epoch basis, attackers returned lower values for TD (-16.74, CI: -27.10 to -6.42 m·min^-1^), LSR (-10.06, CI: -17.23 to -2.90 m·min^-1^), and MSR (-7.67, CI: -12.73 to -2.61 m·min^-1^), compared with midfielders (all p ≤0.05). In addition, defenders performed less TD (-16.28, CI: -29.51 to -3.05 m·min^-1^) and MSR (-9.17, CI: -15.65 to -2.70 m·min^-1^) per epoch than midfielders, while covering less distance (-2.36, CI: -2.52 to -0.01 m) whilst decelerating at moderate-speed (all p ≤0.05). On occasions in which players entered the pitch when the match scores were level, substitutes performed less HSR (-3.39, CI: -6.57 to -0.41 m·min^-1^) and covered less distance whilst decelerating at moderate-speed (-2.60, CI: -3.29 to 0.00 m), per epoch, compared with when the reference team was winning at the time of introduction (all p ≤0.05). Moreover, when a substitute was introduced in a winning scenario, greater moderate-speed deceleration distance (2.32, CI: 0.01 to 2.68 m) was recorded per post-pitch-entry epoch, compared with when the team was behind in the match (p = 0.014).

## Discussion

Considering both the pre- and post-pitch-entry periods, this case study assessed the movement profiles of substitute players from an English professional soccer club following the implementation of a modified match-day pre-pitch-entry protocol. Substitutes performed 3 ± 1 rewarm-up bouts∙player^-1^∙match^-1^ between kick-off and pitch-entry, with all rewarm-ups being shorter and eliciting less absolute TD compared with the whole-team pre-match warm-up. Significant increases in relative values for TD, LSR, MSR, and PL were observed between the first and second bouts of player-led rewarm-up activity performed whilst the match was underway, whereas the new staff-led half-time group rewarm-up elicited the greatest absolute responses of any pre-pitch-entry rewarm-up. Although match scoreline appeared to influence substitution timing, substitutes were typically introduced for ~18 min of match-play, with the initial five min following pitch-entry eliciting greater TD, MSR, and PL values than all subsequent epochs. In contrast, no such decline was observed for HSR, which remained similar to 0–5 min post-pitch-entry values throughout. Acknowledging the limitations of a case study approach, these data may aid applied practitioners when designing specific preparation strategies for substitute soccer players. Specifically, although the influence of other team- and match-specific factors cannot be discounted, this investigation observed potential benefits to specific movement-related key performance indicators when substitutes were included within a new whole-team pre-match warm-up, undertook a supervised half-time rewarm-up, and received ongoing player education about the importance of rewarm-up activities.

It is widely accepted that warming-up may be beneficial for improving physical performance and potentially reducing the risk of injury during subsequent high-intensity activity (for reviews, please see: [13–16]), with elevations in muscle (T_m_) and core (T_core_) temperature likely representing the major mechanistic contributor to the ergogenic effects of an active warm-up undertaken prior to team sport-specific exercise [13, 14, 16]. Indeed, a positive relationship exists between increases in body temperature and improvements in explosive exercise performance, with a 1°C change in T_m_ having been associated with up to a ~2–10% augmentation of muscular power output [13, 29]. During the previous competitive season, substitutes from the same soccer club covered ~1 km at ~37.9 m·min^-1^ during their initial pre-match warm-up [12]; values that fall substantially below the ~1.5 km at ~50.3 m·min^-1^ observed in the current investigation (Table 2). Notably, whereas substitutes formerly conducted much of their pre-match warm-up in isolation from members of the starting team, this case study indicates comparatively heightened warm-up physical responses (i.e., in absolute terms) when an integrated approach was taken (Table 2). These findings suggest that if practitioners wish to increase the absolute physical outputs of substitute players during their initial warm-up, and they are not already doing so, including substitutes within the same activities performed by members of the starting team may represent a viable strategy to achieve this objective. Moreover, the ability to complete a routine warm-up has been identified as a valuable coping mechanism to help maintain task-focus amongst international soccer players [30]. As such, it is plausible that including substitutes within the whole-team pre-match warm-up may have conferred important psychological benefits [12], irrespective of the physical or physiological responses elicited.

Although beneficial for enhancing muscular responses during high-intensity exercise performed shortly thereafter, the ergogenic effects of a pre-match warm-up may not exist *ad infinitum*. Indeed, in thermoneutral environments, progressive decreases in body temperature occur alongside concomitant reductions in muscular performance during the ~10–45 min following cessation of an active warm-up [16, 31–33]. Notably, performing short bouts of rewarm-up activity during prolonged (i.e., ≥15 min) transition periods may help to attenuate body temperature declines and thus maintain team sport-specific physical performance when compared with passive rest [16]. Half-time research has also demonstrated a potential reduction in second-half injury-risk following an active rewarm-up performed during the time separating consecutive exercise bouts [19, 21]. Although the first substitution in the current study was typically made after ~69 min of match-play, this value does not include the ~15 min half-time period, the likelihood of first-half stoppage time, or the fact that the English Football Association pitch-protection policy requires that the pre-match warm-up “shall end no later than 10 minutes before the kick-off time” [27]. Accounting for these additional considerations, it is possible that ~69 min of match-play may have equated to upwards of ~100 min following cessation of the initial warm-up. Given such lengthy delays, there exists the potential for physiological processes (e.g., declines in body temperature) to negatively influence physical performance capacity and possibly increase the risk of injury upon a substitute’s introduction into a match [32]; especially if minimal rewarm-up activity is performed between kick-off and pitch-entry [12].

All participants in the current study undertook a ~13 min staff-led rewarm-up on the pitch at half-time. This differs from previous practices within the same club, whereby substitutes chose the characteristics of any half-time activities (i.e., if any were performed) based upon their own perceived needs [12]. Although it is not possible to definitively comment on the relative merits of these diverging strategies, previous half-time rewarm-ups lasted only ~6 min and elicited ~50% of the TD observed in the current study (Table 2, [12]). Given that T_m_ and T_core_ increase progressively during the initial ~15–20 min of muscular activity [13, 34], it is plausible that when combined with the modified pre-match warm-up and provision of ongoing player education, the longer staff-led half-time rewarm-up may have elicited more pronounced and/or longer lasting physiological responses compared with when players themselves determined the activities performed. Further research will be required to substantiate such suggestions, and to assess the potential implications for physical performance and injury-risk following a player’s introduction into a match. However, it is notable that the current investigation observed greater physical responses for substitutes entering the pitch during the second-half, compared with when previous practice was followed.

Similar to values previously reported [12], substitutes in the current case study performed 3 ± 1 rewarm-up bouts∙player^-1^∙match^-1^ between kick-off and pitch-entry (i.e., including the half-time rewarm-up). Acknowledging that other non-pitch-based and/or static activities may also have been performed, players covered ~62.2 m·min^-1^ to ~107.5 m·min^-1^ during these rewarm-ups, which each lasted for between ~3 min and ~13 min. Notably, because regulations in many soccer competitions require team officials to remain within a designated ‘technical area’ whilst a match is underway [27, 35], the precise characteristics of any rewarm-up activities performed in these scenarios must ultimately be determined by the players themselves. Whereas some practitioners may provide substitutes with firm instructions with regards to the expected timing, content, and/or intensity of rewarm-up activity, a more ‘hands-off’ approach (e.g., providing broad guidelines, or allowing players full autonomy to decide upon their own preparations), such as that taken in the current case study, appears to be more common in professional soccer [5]. All players received ongoing education throughout the season, delivered both orally (in one-to-one consultations and group presentation formats) and via the use of posters, regarding the importance of warming-up prior to exercise. Whilst a causal relationship cannot be directly inferred from the data presented, and not considering the single instance of a fourth player-led rewarm-up, relative TD during self-directed rewarm-ups exceeded the values previously reported by up to ~34% (Table 2), without appearing to negatively affect the post-pitch-entry movement responses observed thereafter [12].

Substitutes performed <2 m·min^-1^ of HSR and ≤0.1 m·min^-1^ of SPR during each warm-up and/or rewarm-up undertaken prior to pitch-entry. Despite the ongoing focus on player education, such relative values reflect the responses observed prior to pre-pitch-entry modification (Table 2, [12]). Speculatively, in addition to the potential influence of regulations restricting the level of input from team staff [27, 35], it is possible that a lack of space may have limited the ability to perform high-speed activities during any rewarm-ups undertaken whilst the match is underway (i.e., during the first-half and/or second-half). Whereas substitutes attained lower peak velocities during their independent pitch-side rewarm-ups compared with values recorded during the initial pre-match warm-up, peak velocity was similar between the initial warm-up and the half-time rewarm-up; both of which were led by team staff and conducted on the pitch. Furthermore, amongst online survey respondents, 74% of applied practitioners either ‘agreed’ or ‘strongly agreed’ that substitutes should be provided with more space within which to perform their pre-pitch-entry preparations [5]. Although the design of modern stadia may often represent a barrier to implementation, many practitioners believed that providing additional space and/or permitting staff to accompany substitutes during their rewarm-up activities, may enable more structured rewarm-ups to be conducted at higher intensities than otherwise; potentially enhancing the efficacy of pre-pitch-entry preparations [5]. Whilst some competitions have sought to make such provisions [36], it remains unclear whether or not the presence of additional personnel (e.g., team coaching staff), and/or larger rewarm-up spaces (that may allow the use of equipment, or facilitate more HSR and/or SPR), might positively influence the preparatory strategies adopted by substitutes prior to pitch-entry, and thus possibly translate into improved performance and reduced injury-risk upon a player’s introduction into a match.

When playing time was divided into five min epochs from the moment of a substitute’s entry onto the pitch, the current study appears to indicate generally greater movement demands (i.e., per epoch) compared with former observations from the same club (Table 4, [12]). Indeed, except for returning similar HSR values to those previously reported during the initial five min period following introduction, substitutes in the current investigation substantially exceeded existing findings in relation to TD (~7–10%) and HSR (~23–24%) for at least the first ~15 min post-pitch-entry, with higher PL values also recorded [12]. Speculatively, it is possible that a shorter average playing time for participants in the current study (i.e., the mean timing of introduction for the first, second and third substitutions being ~69 min, ~78 min, and ~86 min, respectively, compared with ~59 min, ~71 min, and ~77 min, respectively) may be somewhat responsible for such data. In support, team sport players may employ conscious or subconscious self-pacing strategies, based upon the anticipated end-point of exercise, producing relatively greater physical outputs for tasks expected to be shorter in duration [7, 37, 38]. However, preliminary analysis indicated that substitution timing (i.e., when categorised as ‘early’, ‘medium’, or ‘late’ substitutions according to 15 min match epochs) did not influence any post-pitch-entry physical performance indicator when assessed on a per epoch basis. Substitution timing was not included in the final linear mixed models due to its consistent non-significance and the fact that it did not improve the model fit based upon either ‘Akaike information criterion’ or ‘Bayesian information criterion’ assessments. It is also possible that modification to pre-pitch-entry preparations may have contributed to the differences in post-pitch-entry responses between studies. Practitioners have highlighted substantial uncertainty as to whether substitutes’ match-day preparations promote optimal readiness for match-introduction [5], and this case study reports the responses following deliberate modification to substitutes’ pre-pitch-entry protocols. Acknowledging the absence of mechanistic data, it is plausible that substitutes in the current investigation were better physically and/or psychologically prepared to produce greater movement responses upon pitch-entry compared with existing practice.

For players who start a match, the highest relative running demands (e.g., TD, HSR, etc.) are typically recorded during the opening ~10–15 min of play [6, 7]. Moreover, it has been suggested that such elevated initial physical outputs may be at least partly specific to the time of pitch-entry for any given individual, rather than necessarily the proximity to match kick-off [12]. In the current case study, although TD, LSR, MSR, and PL decreased between 0–5 min and 5–10 min post-pitch-entry, physical outputs were maintained to a greater extent than previously (Table 4). For example, whereas existing research highlighted ~12%, ~31%, and ~39% reductions in TD, MSR, and HSR, respectively [12], the between-epoch decreases following pre-pitch-entry modification were ~10% for TD and ~17% for MSR, while HSR remained similar to 0–5 min values throughout (Table 4). Whilst the match-specific consequences of these responses remain unclear, a substitute’s ability to make an immediate and sustained physical impact upon pitch-entry is highly valued amongst practitioners [5], and supports the playing philosophy of the club recruited in the study (empirical evidence).

When averaged across match epochs (i.e., per epoch), substitute midfielders covered an additional ~17 m·min^-1^ and ~16 m·min^-1^ compared with attackers and defenders, respectively. These findings are consistent with a body of literature indicating that amongst both whole- and partial-match players, midfielders typically cover the greatest relative distances of any playing position and may suffer the greatest between-half declines in physical outputs [10–12, 39]. Acknowledging that the use of substitutions can also reflect several other objectives [3, 5], for coaches/managers seeking to maintain physical output across a team, this phenomenon could suggest a benefit to replacing midfielders during the second-half of match-play, and perhaps partly explains why midfielders represent the position most often substituted in professional soccer [3, 4, 40]. Due to sample size considerations, it was not possible to compare physical responses between sub-categories of each position. However, despite the sample containing six of the same players and reflecting a broadly similar positional profile to that recruited previously (i.e., 18, 11, and six observations from midfielders, attackers and defenders, respectively, compared with the 16, 14, and five observations previously reported for the same positional groups [12]), there exists the potential that differences in the ‘style’ of players sampled may have influenced the physical responses observed following pitch-entry. Future research investigating substitutes’ positional responses in greater detail may provide valuable information for informing substitution strategies.

Although substitutes typically spent ~18 min on the pitch, the average for players introduced when the team was ahead in the match was just ~14 min. In contrast, ~32 min and ~23 min were played by individuals who entered the pitch when the reference team was either losing or drawing, respectively. Alongside highlighting the need for players entering the pitch to be appropriately conditioned to provide a sustained impact for upwards of ~30 min of match-play, such observations confirm that scoreline may represent an important factor influencing the timing of substitutions [3, 4]; apparently indicating a greater willingness for teams to make tactical changes when they are behind in a match. Indeed, as the ultimate objective of soccer is to outscore the opposition, it seems likely that coaches and managers typically value the role of substitutes more highly at times when their team is losing compared with when the players already on the pitch have managed to produce a lead. Match scoreline may also influence a player’s running demands, and this study reflects existing observations that indicated how substitutes covered the greatest TD and/or HSR per epoch when the reference team was winning at the time of pitch-entry [12]. Compared with previous research in which the team was leading at the time of the ~37% of substitutions made, the fact that ~69% of substitutions in the current investigation occurred in winning scenarios could at least partly explain the between-study differences in substitution timing and may also have contributed to elevating the physical outputs of players entering the pitch.

Between the timing of a player’s pitch-entry and the end of the match, the team goal differential improved following 26% of the substitutions observed. As this percentage is identical to that reported previously [12], it seems that modification of pre-pitch-entry practices was not detrimental to this crucial marker of team performance. Moreover, such values occurred in the current case study with substitutes having been introduced on average ~7–10 min later in the match. Notably, whereas previous research reported a worsening scoreline following 20% of substitutions [12], this figure was almost halved (i.e., 11%) in the present investigation. Acknowledging that such findings may be attributable to a range of contextual factors (e.g., the relative quality of the opposition, team tactics, the performance of players already on the pitch, etc.), these patterns indicate more favourable team responses following the introduction of substitutes undertaking the modified practices presented here, when compared with the outcomes previously reported.

The findings of this case study highlight several avenues for future investigation. Whilst analysing a player’s movement profiles provides valuable insight into the preparatory practices undertaken, controlled research determining the physiological (e.g., body temperature etc.) and performance (i.e., physical, technical, cognitive, etc.) responses of substitutes prior to pitch-entry would allow greater comment on the efficacy of such strategies; thereby facilitating the design of specific interventions targeted at optimising a player’s readiness for match-introduction. Moreover, investigation into the effects of certain regulatory conditions (e.g., pitch-protection policies, restrictions on staff involvement with rewarm-up activity, etc.) may help policy-makers to reach fully informed decisions when defining the terms of competition legislation. Nevertheless, this case study observed heightened HSR and peak velocity values during the staff-led half-time group rewarm-up compared with those recorded during numerous player-directed pitch-side rewarm-ups. Such responses may suggest that the presence of team staff during rewarm-ups and/or the availability of space within which to perform HSR could represent important factors influencing the physical preparations of awaiting substitutes.

Practitioners value the introduction of ‘fresh legs’ as a means of providing a physical impact upon a match [5], and the amount of HSR performed represents an important indicator of physical performance in soccer [6, 9]. However, whilst useful for quantifying the locomotor demands experienced on match-day, MEMS data in isolation cannot determine whether a period of heightened activity in fact represents a useful contribution to team success. Although it is notable that favourable match scoreline responses were observed compared with those previously reported, future research into the specific tactical impact of substitutions would be beneficial for informing team strategy. Indeed, taking an integrated approach in combining physical, technical, and tactical indices of match performance, may allow more holistic assessment of a substitute’s value [41].

## Conclusion

On match-day, substitutes from a professional soccer club performed a pre-match warm-up followed by ~3 bouts·player·match^-1^ of rewarm-up activity between kick-off and pitch-entry. After involvement in a previous study [12], the club implemented a club-wide strategy leading to a modification of substitutes’ pre-pitch-entry practices. Modification involved the combined intervention of, substitutes; completing an amended pre-match warm-up alongside members of the starting team, performing ~13 min of staff-led activity on the pitch at half-time, and receiving an ongoing educational programme focusing on the importance of (re)warm-up activity as preparation for match-play. Although a direct causal link cannot be established from the data presented in the current case-study, this investigation observed generally heightened movement responses in substitutes before and after pitch-entry, compared with those previously reported. Furthermore, alongside equivalent rates of improvement in team goal differential (i.e., following 26% of substitutions observed) despite players having been introduced later in the match, the incidence of a worsening scoreline following a substitution was almost halved (i.e., 11% versus 20%) in the present investigation.

Future research into the specific physiological responses of partial-match players will be important to help practitioners seeking to optimise the match-day preparations of this unique playing population. Notably, regulatory and/or practical considerations may represent a barrier to rewarm-up activity; factors that could contribute to the limited amount of HSR and/or SPR performed prior to pitch-entry. Once introduced into a match, substitutes covered ~122 m·min^-1^ during their ~18 min playing bout, with substantial reductions in TD between the first and second five min epochs following pitch-entry. However, values generally remained higher than those reported previously, and no significant between-epoch decline was observed for HSR. Investigation into the tactical impact of making a replacement would provide further valuable insight, while quantifying within- and post-match fatigue responses may help to inform the design of preparatory and recovery strategies based upon the unique match-day demands faced by substitutes.

## Supporting information

S1 Data (XLSX)
